# Cardiac resynchronisation therapy in optima forma

**DOI:** 10.1007/s12471-022-01682-y

**Published:** 2022-04-07

**Authors:** S. A. J. Timmer, T. Germans

**Affiliations:** Department of Cardiology, Northwest Clinics, Alkmaar, The Netherlands

A 60-year-old male with heart failure and severely reduced left ventricular function was scheduled for elective implantation of a cardiac resynchronisation therapy defibrillator device. Coronary artery angiography revealed no significant coronary artery disease. He remained symptomatic despite guideline-directed optimal medical therapy. The ambulatory electrocardiogram (ECG) showed sinus rhythm with a slightly prolonged PR interval, ventricular ectopic beats and complete left bundle branch block (LBBB) with QRS duration ~150 ms and mid-QRS notching in > 2 leads, consistent with the Strauss criteria for LBBB (Fig. 1; [1]). The device implantation was successful, and there were no procedural complications. A postoperative ECG was obtained on the cardiology ward shortly after the procedure (Fig. 2).

**Fig. 1:**
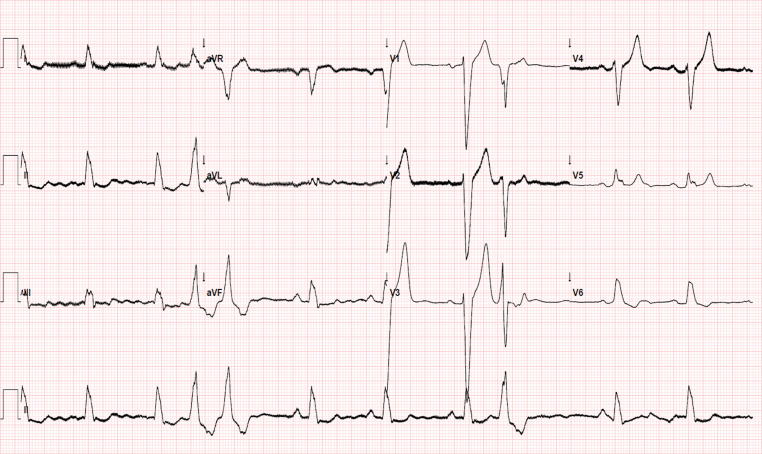
Preoperative electrocardiogram

**Fig. 2:**
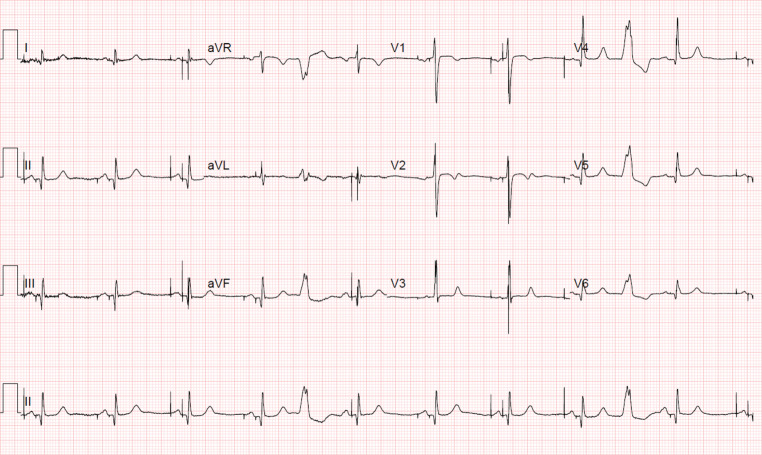
Postoperative electrocardiogram

What type of pacing can be seen, and what other distinctive pathology is revealed?

## Answer

You will find the answer elsewhere in this issue.
